# Rehabilitation of Enucleated Eye by Ocular Prosthesis: Role of Parents in Prosthesis Development

**Published:** 2012-09-01

**Authors:** Jitendra Rao, Lakshya Kumar, Pradeep Kumar

**Affiliations:** Dept.of Prosthodontics, Faculty Of Dental Sciences, CSMMU (Erstwhile KGMC), Lucknow, Uttar Pradesh India- 226001.

**Dear Sir**

The disfigurement resulting from loss of an eye can cause significant psychological as well as social consequences for both the patient and family. Ambroise Pare, a French surgeon-dentist, is considered the pioneer of modern artificial eyes. In 1944 Murphy and Nirronen fabricated physiologic ocular prosthesis in dental corps of US Navy [1]. An ocular prosthesis does not provide vision; but give psychological support and cosmesis. The scleral shell prosthesis is a thin hard acrylic shell-like artificial eye. This type of eye prosthesis is worn over a damaged, disfigured eye or eviscerated globe. It provides psychological support to patient and his family.

A 3-year-old female child was referred to department of Prosthodontics with the history of enucleated left eye two month back due to retinoblastoma of eye (Fig. 1). Keeping in mind the aesthetics and age of the patient, it was planned to make an ocular prosthesis. First, petroleum jelly was applied to the eye-brows for the easy removal of the impression after setting. For impression a customized perforated tray was used (Fig. 2). A thin mix of ophthalmic alginate (Ophthalmic moldite, Milton Roy Co. Sarasota Fla.) was used. The patient was asked to move her normal eye in all directions to allow the alginate to flow into all areas of the enucleated socket. The technique was modified here onwards by trimming out the iris portion of the stock eye and orienting it on the cast according to previously transferred pupillary mark. Tooth-colored acrylic (SC 10, Pyrax, Roorkee, India) was matched with the color of sclera of the opposite eye. Then the adjusted and modified stock eye-wax pattern combination was processed. Red silk fibres to mimic veins were placed in the dough of the determined acrylic shade followed by routine curing, finishing and polishing. Finally, a thin film of the sclera was removed and replaced by a clear film of transparent heat-cured PMMA (Trevalon, Dentsply India Pvt. Ltd., Gurgaon, India). The properly finished and polished prosthesis was inserted in the socket after being disinfected and lubricated with an ophthalmic lubricant (Ecotears, Intas Pharmaceuticals Ltd, Ahmedabad, India) (Fig 3). Necessary instructions for cleaning, insertion and removal of the prosthesis were given to the parents. 

**Figure:**
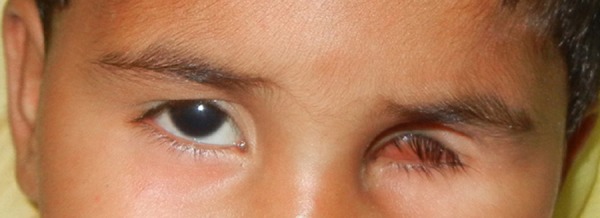
Figure 1: Clinical presentation of the defect.

**Figure:**
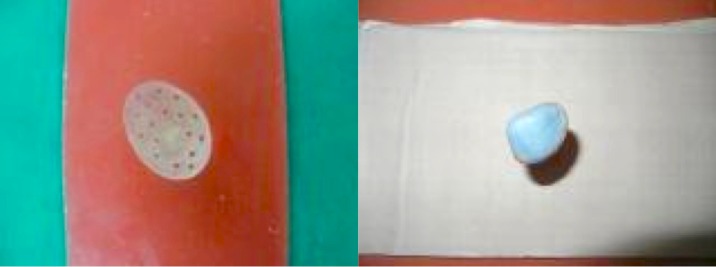
Figure 2: Acrylic shell for impression and impression of defect.

**Figure:**
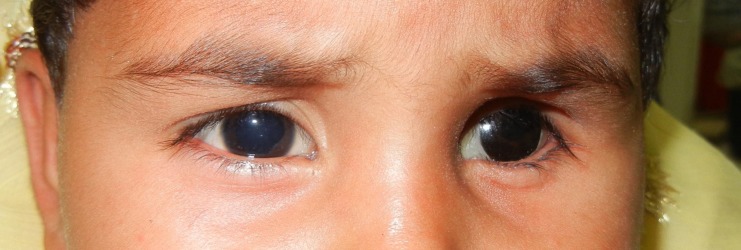
Figure 3: Eye Prosthesis inserted.

Active participation of parents, motivation and adjustment while prosthodontic procedure completed, is necessary until the treatment is completed. Fabrication of aesthetic orbital prosthesis poses more difficulty than routine cases especially for an emotionally vulnerable child. In this situation, parent’s cooperation is much more expected and needed [2]. Treatment planning can be modified as hydroxyappatite integrated ocular implant developed by Perry [3] but it was not indicated for paediatric cases. Sykes used medium viscosity polyvinyl siloxane impression material. A modification of the technique described by Taicher et al was performed by Sykes [4]. An Impression of the socket in ophthalmic irreversible hydrocolloid in conjunction with a suitable impression tray is preferred due to ease and better control of the procedure. Parents are guided to encourage the child during each procedure especially during impression procedure to facilitate the uniform flow of material inside the socket. As the patient was very uncooperative parents were trained and guided properly to achieve the optimum results in all steps of the treatment phase.

## Footnotes

**Source of Support:** Nil

**Conflict of Interest:** None declared
